# Independent and Combined Effects of Physical Activity and Sedentary Behavior on Blood Pressure in Adolescents: Gender Differences in Two Cross-Sectional Studies

**DOI:** 10.1371/journal.pone.0062006

**Published:** 2013-05-01

**Authors:** Augusto César Ferreira de Moraes, Heráclito Barbosa Carvalho, Juan Pablo Rey-López, Luis Gracia-Marco, Laurent Beghin, Anthony Kafatos, David Jiménez-Pavón, Dénes Molnar, Stefaan De Henauw, Yannis Manios, Kurt Widhalm, Jonatan R. Ruiz, Francisco B. Ortega, Michael Sjöström, Angela Polito, Raquel Pedrero-Chamizo, Ascensión Marcos, Frederic Gottrand, Luis A. Moreno

**Affiliations:** 1 Department of Preventive Medicine, School of Medicine of the University of São Paulo, São Paulo, SP, Brazil; 2 GEPECIN–Science of Nutrition Group Research, Pontifícia Universidade Católica do Paraná (PUCPR), Paraná, Brazil; 3 GENUD–Growth, Exercise, Nutrition and Development, University of Zaragoza, Zaragoza, Spain; 4 School of Health Sciences of the University of Zaragoza, Zaragoza, Spain; 5 Unité Inserm U995 and Université Lille Nord de France, Lille, France; 6 Preventive Medicine and Nutrition Unit, University of Crete School of Medicine, Heraklion, Crete, Greece; 7 PROFITH Group, Department of Physical Education and Sports, School of Sport Sciences, University of Granada, Granada, Spain; 8 Department of Paediatrics, Medical Faculty, University of Pécs, Pécs, Hungary; 9 Department of Public Health, Ghent University, Ghent, Belgium; 10 Department of Nutrition and Dietetics, Harokopio University, Athens, Greece; 11 Division of Nutrition and Metabolism, Department of Pediatrics, Medical University of Vienna, Vienna, Austria; 12 Department of Biosciences, Unit for Preventive Nutrition, Karolinska Institutet, Stockholm, Sweden; 13 National Institute for Food and Nutrition Research, Rome, Italy; 14 Universidad Politecnica de Madrid, Health and Human Performance, Madrid, Italy; 15 Immunonutrition Research Group, Department of Metabolism and Nutrition, Food Science and Technology and Nutrition Institute, Spanish National Research Council, Madrid, Spain; 16 School of Sport and Health Sciences, University of Exeter, Exeter, United Kingdom; 17 Centre d′Investigation Clinique, CIC-9301-Inserm-CH&U, Lille, France; 18 Department of Preventive Medicine, School of Medicine of the University of São Paulo, São Paulo, SP, Brazil; 19 GICRAF–Scientific Research Group Related to Physical Activity, UNESP, Presidente Prudente, SP, Brazil; Universidade Federal do Rio de Janeiro, Brazil

## Abstract

**Objectives:**

To examine the independent and combined association of physical activity (PA) and sedentary behavior (SB) on both systolic (SBP) and diastolic blood pressure (DBP) in adolescents from two observational studies.

**Methods:**

Participants from two cross-sectional studies, one conducted in Europe (n = 3,308; HELENA study) and the other in Brazil (n = 991; BRACAH study), were selected by complex sampling. Systolic and diastolic blood pressure (outcomes), PA and SB, both independently and combined, and potential confounders were analyzed. Associations were examined by multilevel linear regression.

**Results:**

Performing the recommended amount of PA (≥60 min/d) attenuated the effect of SB on DBP in BRACAH study girls and in boys from both studies. In contrast, PA did not attenuate the effects of SB on the SBP of girls in the HELENA study. The combination of less than recommended levels of PA with 2–4 h/d of sedentary behavior was found to be associated with increased SBP in boys from both studies.

**Conclusions:**

Meeting current PA recommendations could mediate the association between SB and DBP in both sexes. In boys, the joint effect of low levels of PA and excessive sedentary activity increases SBP levels. Longitudinal studies are required to confirm these findings.

## Introduction

Chronic non-communicable diseases (NCDs) are the main source of disease burden worldwide and are thus a major public health problem [1]. Among NCDs, hypertension has been shown to have the highest prevalence in adults [2], and studies have shown that blood pressure (BP) levels in childhood and adolescence greatly impact the development of hypertension in adulthood [3].

Among the factors that may influence blood pressure levels (e.g. genetics, intrauterine development, socioeconomic status, tobacco use, waist circumference, obesity), patterns of physical activity (PA) and sedentary behavior (SB) have been shown to have inverse [4] and direct associations,[5], [6] respectively, with blood pressure in adolescents.

Although the effects of PA and SB on BP have mainly been examined in isolation, there are studies suggesting that these behaviors have an aggregate effect on adolescents [7], [8]; however, few studies have quantified the association between joint PA/SB levels and blood pressure in adolescents [7], [8]. On the other hand, PA/SB levels are associated with sociodemographic and economic variables. The influence of sociodemographic factors on PA/SB bas been described in a review [9]. There is no consensus in the literature regarding socioeconomic variables as determinants of these behaviours since such differences may be attributed to the demographic context and characteristics of the populations studied rather than the individual [10], [11]. For this reason, we have included results from a multi-national European study and another one conducted in South America (Brazil) in this report.

Reproducing the same results in different population groups with different characteristics would increase their biological plausibility and provide a higher level of scientific evidence. For this reason, we tested the hypothesis, separately, in two cross-sectional studies conducted with adolescents: Healthy Lifestyle in Europe by Nutrition in Adolescence (HELENA) and Brazilian Cardiovascular Adolescent Health (BRACAH).

Thus, we hypothesized that higher levels of PA would attenuate the adverse effect of high SB levels on BP and that the combined effect of low PA and high SB levels may contribute to increased BP levels and that these effects may be different depending on where adolescents live.

## Methods

The HELENA study was based on data from a random sample of European adolescents who were tested on a wide range of nutrition and health-related parameters. The data were collected in 2006 and 2007 in ten cities from nine European countries. A detailed description of the HELENA sampling and recruitment methodology, harmonization processes, data collection, analysis strategies and quality control activities has been published elsewhere [12]. After receiving complete information about the aims and methods of the study, all parents/guardians signed an informed consent form and the adolescents agreed to participate in the study. The protocol was approved by the Human Research Review Committees of the centers involved.

Data from the BRACAH study were collected in 2007 in the city of Maringá, PR, Brazil, population approximately 330,000 (51,428 adolescents, 50.1% female). The adolescents were selected by random sample and evaluated on a broad range of cardiovascular risk factors and various health behavior parameters. The complete sample size methodology of this study has been described previously [13]. A formal request to conduct this survey was submitted to and accepted by the boards of several public and private schools. This study was also approved by the Ethics Committee on Research Involving Human Participants of the University Center of Maringá and authorized by the Ethics Committee on Research Projects of the University of São Paulo in accordance with Brazilian laws.

For the current study, we selected adolescents from HELENA and BRACAH with complete data regarding gender, age, systolic BP (SBP), diastolic BP (DBP) (outcomes), PA levels, SB, socioeconomic status, parental education, regular tobacco consumption, body mass index and waist circumference. These variables are described in detail below.

A total of 3,308 adolescents from the HELENA study (12.5–17.5 years old) and 991 adolescents from the BRACAH study (14.0–17.5 years old) met all the inclusion criteria and were included in the analyses.

### Blood Pressure Measurements

In both studies, BP measurements were performed following the recommendations for adolescent populations [14]. In both studies BP was measured twice after weight and height measurements were taken. The subjects were seated in a separate, quiet room for 10 min with their backs supported and feet on the ground. Two BP readings were taken with a 10 min interval of quiet rest. The lower of the two measurements was used.

Systolic and Diastolic BP were measured by the arm blood pressure oscillometric monitor device OMRON® M3 (HEM 742) in the BRACAH study and the OMRON® M6 (HEM 70001) in the HELENA study. The OMRON® M3 (HEM 742) has been clinically and epidemiologically validated for adolescents by the Brazilian Research Group[15]. The OMRON® M6 (HEM 70001) has been approved by the British Hypertension Society [16]. These data collection procedures have been described in an earlier study [17].

### Independent variables

The PA and SB levels were considered independent variables and measured by means of questionnaires in both studies. The questionnaire model used for PA measurements in both studies was developed to assess PA levels (moderate-to-vigorous levels) in adolescents [18].

In the HELENA study, PA was also measured with accelerometers (Actigraph MTI, model GT1M, Manufacturing Technology Inc., Fort Walton Beach, FL, USA) for seven consecutive days, with a minimum of 8 hours recording/day for at least 3 days [19]. The time sampling interval (epoch) was set to 15 seconds. Inactive, moderate and vigorous PA was defined as <100, 2000–3999 and ≥4000 counts per minute, respectively. The cutoffs selected were similar to those used in previous studies [20], [21]. In both methodologies (questionnaire and accelerometry), and following current PA guidelines, [22], [23] subjects were classified as active when they accumulated at least 60 min/d of moderate-to-vigorous PA.

Sedentary behavior levels were assessed with a structured questionnaire, including questions on time habitually spent in front of the television, the computer and/or playing video games. In both studies, the questionnaire used questions such as "*During weekdays, how many hours do you usually spend watching television*?"-"*During weekdays, how many hours do you usually spend on computers*?-"*During weekdays, how many hours do you usually spend playing video games*?" Sedentary behavior was totaled and classified into the following categories: 0–2 h/d; >2–4 h/d; ≥4 h/d according to Dunstan et al. [24]. The same questions were asked for weekend days and this questionnaire was used with adolescents from both studies as a realiability, validity and translated tool [25]–[27].

We also established six clusters of PA according to PA recommendations [22], [23] and SB according to Dunstan et al. [24] for use with both measurement methods, which are described below.

Questionnaire:

1. <60 min/d of PA +>4 h/d of SB;
2. <60 min/d of PA +2–4 h/d of SB;
3. <60 min/d of PA +<2 h/d of SB;
4. ≥60 min/d of PA +>4 h/d of SB;
5. ≥60 min/d of PA +2–4 h/d of SB;
6. ≥60 min/d of PA +<2 h/d of SB;

Accelerometer (using PA recommendations and tertiles of sedentary time):

1. <60 min/d of PA of PA +3^rd^ tertile of SB;
2. <60 min/d of PA +2^nd^ tertile of SB;
3. <60 min/d of PA +1^st^ tertile of SB;
4. ≥60 min/d of PA +3^rd^ tertile of SB;
5. ≥60 min/d of PA +2^nd^ tertile of SB;
6. ≥60 min/d of PA +1^st^ tertile of SB;

### Potential confounders

The potential confounders for this study were:

  Country (HELENA only):
  Age (years):
  Socioeconomic status: based on the family's household goods. In the HELENA study, the same definitions were used in previous HELENA studies [28], [29]. In the BRACAH study, the Brazil Criterion of Economic Classification [30] was employed. Three levels were used to classify socioeconomic status: low, medium and high.
  Parental education: determined with a self-reported questionnaire and classified into four levels: elementary education, lower secondary education, upper secondary education and university degree.
  Regular tobacco smoking: defined as the regular consumption of at least one cigarette per day for a minimum of one month [31];
  Body mass index (BMI): calculated as weight (kg)/height(m^2^). BMI was used as a continuous variable in the analysis. Wearing light clothes and no shoes, the adolescents' height was measured to the nearest 0.1 cm with a wood stadiometer and their body mass to the nearest 0.1 kg with a calibrated portable digital scale.
  Waist circumference: measured in both studies at the midpoint between the lowest point of the rib cage and the top of the iliac crest next to skin with a non-elastic measuring tape to the nearest 0.1 cm.

### Statistical Analysis

The descriptive analyses were presented as means (quantitative variables), percentages (qualitative variables) and 95% confidence intervals (CI95%). Multilevel linear regression models using fixed effects intercept were fitted to analyze the relationship between each BP level and independent variables [32], [33], considering two levels of data organization: (i) individual behaviors and (ii) potential confounders (not shown) [34]. The context variable used was the school. Homoscedasticity was graphically assessed in all regression models to meet the analysis criteria. *p*-values of ≤0.20 were adopted in the univariate analysis [34] since they were necessary to include variables in the multivariate analysis and then the hierarchical model method according to the above-mentioned levels. *P*-values <0.05 or those representing >10% modification in the β of any variable already in the model were considered significant.

The multilevel analyses were performed with two objectives: 1^st^) to test the associations between BP and two separate measures of individual behavior; 2^nd^) to test the extent to which country-specific characteristics and contextual variables mediate the associations between SBP and DBP levels and PA and SB.

Stata 12 (Stata Corp., College Station, TX, USA) was used for all statistical calculations. All analyses were adjusted for the clustered nature of the sample using the "svy" set of commands and stratified by gender, since interactions between sex and the studied variables were observed (*p*<0.001).

For adolescents from the HELENA study (boys  = 1,106; girls  = 960) we conducted a comparative analysis between the PA and SB levels found with the questionnaires and the PA measures found with and without the use of accelerometers. No significant differences were found for either sex (p = 0.406 for boys and p = 0.714 for girls).

## Results

Subject characteristics, sociodemographic/socioeconomic variables, BP levels and PA and SB levels (independent and combined) are shown in a supplementary file. Table 1 shows the β coefficients from multilevel linear regression for DBP in girls. Table 2 shows the association between PA and SB patterns and DBP for boys. There was a positive association between those adolescents who did not meet the PA guidelines and DBP in the HELENA study. However, these associations were not significant after adjusted analysis (Table 2).

**Table 1:** Multiple linear regression analysis evaluating the association between diastolic blood pressure according to independent variables for each study, in girls.

Independents Variables	Null model		Unadjusted		Adjusted*	
	HELENA	BRACAH	HELENA	BRACAH	HELENA	BRACAH
	ß (95% CI)	ß (95% CI)	ß (95% CI)	ß (95% CI)	ß (95% CI)	ß (95% CI)
**Fixed Effects Constant**	68.5 (68.1–68.9)	67.1 (66.2–68.0)			56.6 (51.2–61.9)	78.9 (69.1–88.8)
**Physical activity by questionnaire**			p = 0.894	p = 0.011	p = 0.737	p = 0.123
≥60 min/d			Ref	Ref	Ref	Ref
<60 min/d			0.08 (−1.06–1.21)	**2.56 (0.59 –4.53)**	−0.18 (−1.58–1.23)	1.68 (−0.46–3.82)
**Sedentary behavior by questionnaire**			p = 0.066	p<0.001	p = 0.54	p = 0.501
<2 h/d			Ref	Ref	Ref	Ref
2–4 h/d			0.41 (−0.57–1.39)	**10.49 (4.83–16.15)**	−0.02 (−1.18–1.14)	3.01 (−3.13–9.15)
>4 h/d			1.10 (−0.06–2.26)	**13.31 (8.15–18.47)**	0.37 (−1.03–1.78)	2.83 (−2.75–8.41)
**Physical activity by accelerometers** **			p = 0.159		p = 0.281	
<60 min/d			Ref		Ref	
≥60 min/d			0.79 (−0.31–1.89)		0.72 (−0.52–2.04)	
**MPA by accelerometers (min/d)**			p = 0.499		p = 0.765	
			0.45 (−0.86–1.76)		−0.01 (−0.05–0.03)	
**VPA by accelerometers (min/d)**		p = 0.824		p = 0.163		
			−0.01 (−0.05–0.04)		−0.04 (−0.09–0.02)	
**MVPA by accelerometers (min/d)**		p = 0.41		p = 0.342		
			−0.04 (−0.10–0.01)		−0.01 (−0.04–0.02)	
**SB by accelerometers (min/d)**		p = 0.707		p = 0.544		
			−0.01 (−0.02–0.01)		0.01 (−0.01–0.01)	
**Cluster PA and SB by accelerometers** *		p = 0.265		p = 0.415		
<60 min/d+3° tercil			Ref		Ref	
<60 min/d+3^rd^ tertile			−0.05 (−1.65–1.56)		−0.15 (−1.79–1.49)	
<60 min/d+2^nd^ tertile			−0.26 (−2.07–1.55)		−0.35 (−2.19–1.50)	
<60 min/d+1^st^ tertile			−0.04 (−2.53–2.45)		−0.16 (−2.81–2.48)	
≥60 min/d+3^rd^ tertile			−1.24 (−3.41–0.92)		−1.52 (−3.79–0.74)	
≥60 min/d+2^nd^ tertile			−0.80 (−2.95–1.36)		−0.29 (−2.56–1.98)	
**Intraclass correlation coeficient**	0.09	0.02			0.09	0.06
**Standard deviation context**	2.74	1.62			3.88	2.68
**Standard deviation individual**	8.74	10.41			9.88	10.96
**Akaike Information Criterion**	11,929.8	4,043.2			11,084.5	4,116.5

Beta coefficient and their respective confidence intervals 95% (95% CI).

* This analysis was adjusted for potential confounders: *age, socioeconomic status, parental education, regular tobacco smoking, body mass index and waist circumference.*

** 622 girls were excluded because they did not meet the inclusion criteria.

MPA = moderate physical activity.

VPA = vigorous physical activity.

MVPA = moderate and vigorous physical activity.

PA = physical activity.

SB = sedentary behavior.

Significant associations are in bold.

**Table 2:** Multiple linear regression analysis evaluating the association between diastolic blood pressure according to independent variables for each study, in boys.

Independents Variables	Null model		Unadjusted		Adjusted*	
	HELENA	BRACAH	HELENA	BRACAH	HELENA	BRACAH
	ß (95% CI)	ß (95% CI)	ß (95% CI)	ß (95% CI)	ß (95% CI)	ß (95% CI)
**Fixed Effects Constant**	67.8 (67.4–68.3)	68.9 (68.1–69.9)			58.7 (52.9–64.4)	44.7 (35.9–53.4)
**Physical activity by questionnaire**			p = 0.002	p = 0.178	p = 0.012	p = 0.178
≥60 min/d			Ref	Ref	Ref	Ref
<60 min/d			**2.02 (0.73–3.30)**	1.41 (−0.65–3.46)	**2.01 (0.43–3.59)**	1.43 (−0.65–3.51)
**Sedentary behavior by questionnaire**			p = 0.312	p = 0.575	p = 0.477	p = 0.671
<2 h/d			Ref	Ref	Ref	Ref
2–4 h/d			**1.29 (0.06–2.52)**	−0.03 (−4.68–4.63)	1.80 (−0.34–3.26)	0.57 (−4.15–5.30)
>4 h/d			0.83 (−0.40–2.52)	0.62 (−3.76–5.00)	0.81 (−0.67–2.29)	0.87 (−3.56–5.30)
**Physical activity by accelerometers** **			p = 0.575		p = 0.475	
<60 min/d			Ref		Ref	
≥60 min/d			0.37 (−0.94–1.70)		0.50 (−0.55–0.95)	
**MPA by accelerometers (min/d)**			p = 0.767		p = 0.952	
			−0.01 (−0.05–0.04)		0.01 (−0.04–0.05)	
**VPA by accelerometers (min/d)**		p = 0.487		p = 0.647		
			−0.02 (−0.06–0.03)		−0.01 (−0.06–0.03)	
**MVPA by accelerometers (min/d)**		p = 0.566		p = 0.832		
			−0.01 (−0.03–0.02)		−0.01 (−0.03–0.02)	
**SB by accelerometers (min/d)**		p = 0.729		p = 0.942		
			0.01 (−0.01–0.01)		0.01 (−0.01–0.02)	
**Cluster PA and SB by accelerometers** *		p = 0.681		p = 0.455		
<60 min/d+3° tercil			Ref		Ref	
<60 min/d+3^rd^ tertile			−(0.29 (2.73–215)		−0.82 (−3.34–1.96)	
<60 min/d+2^nd^ tertile			−0.08 (−2.51–2.34)		−0.29 (−2.77–2.19)	
<60 min/d+1^st^ tertile			0.48 (−1.91–2.86)		0.29 (−2.16–2.76)	
≥60 min/d+3^rd^ tertile			−1.99 (−4.17–0.18)		−2.19 (−4.42–0.03)	
≥60 min/d+2^nd^ tertile			0.17 (−1.82–2.16)		−0.15 (−2.27–1.97)	
**Intraclass correlation coeficient**	0.10	0.07			0.13	0.05
**Standard deviation context**	3.46	2.54			3.96	2.09
**Standard deviation individual**	10.53	9.51			10.27	9.33
**Akaike Information Criterion**	11,533.8	3286.2			10,324.3	3,042.6

Beta coefficient and their respective confidence intervals 95% (95% CI).

* This analysis was adjusted for potential confounders: *age, socioeconomic status, parental education, regular tobacco smoking, body mass index and waist circumference.*

** 420 boys were excluded because they met the inclusion criteria.

MPA = moderate physical activity.

VPA = vigorous physical activity.

MVPA = moderate and vigorous physical activity.

PA = physical activity.

SB = sedentary behavior.

Significant associations are in bold.

A positive and significant association was found for HELENA study girls, after adjustment for confounding variables, between ≥60 min/d of PA+3^rd^ tertile of SB and systolic BP (Table 3). The association between PA and SB patterns and SBP for boys is presented in Table 4. No independent variables showed significant association after adjustment for confounding variables.

**Table 3:** Multiple linear regression analysis evaluating the association between systolic blood pressure according to independent variables for each study, in girls.

Independents Variables	Null model		Unadjusted		Adjusted*	
	HELENA	BRACAH	HELENA	BRACAH	HELENA	BRACAH
	ß (95% CI)	ß (95% CI)	ß (95% CI)	ß (95% CI)	ß (95% CI)	ß (95% CI)
**Fixed Effects Constant**	117.6 (115.7–119.6)	107.0 (101.1–108.0)			90.4 (83.4–97.5)	77.2 (66.9–87.5)
**Physical activity by questionnaire**			p = 0.844	p = 0.054	p = 0.443	p = 0.099
≥60 min/d			Ref	Ref	Ref	Ref
<60 min/d			−0.15 (−1.62–1.33)	2.11 (−0.04–4.27)	−0.64 (−2.38–1.11)	1.63 (−0.50–3.77)
**Sedentary behavior by questionnaire**			p = 0.130	p = 0.402	p = 0.963	p = 0.501
<2 h/d			Ref	Ref	Ref	Ref
2–4 h/d			0.43 (−0.83–1.70)	4.05 (−2.30–10.39)	−0.20(−1.63–1.24)	3.47 (−2.65–9.61)
>4 h/d			1.53 (0.04–3.03)	3.95 (−1.83–9.73)	0.05 (−2.39–1.82)	2.98 (−2.59–8.55)
**Physical activity by accelerometers** **			p = 0.635		p = 0.818	
≥60 min/d			Ref		Ref	
<60 min/d			0.40 (−2.08–1.27)		0.20 (−1.99–1.53)	
**MPA by accelerometers (min/d)**			p = 0.921		p = 0.706	
		0.02 (−0.01–0.06)		−0.01 (−0.07–0.05)		
**VPA by accelerometers (min/d)**			p = 0.16		p = 0.176	
		−0.05 (−0.12–0.02)		−0.05 (−0.13–0.02)		
**MPA by accelerometers (min/d)**			p = 0.484		p = 0.364	
		−0.01 (−0.05–0.02)		−0.02 (−0.06–0.02)		
**SB by accelerometers (min/d)**			p = 0.328		p = 0.331	
		0.01 (−0.01–0.01)		0.01 (−0.01–0.01)		
**Cluster PA and SB by accelerometers** *			p = 0.876		p = 0.979	
<60 min/d+3^rd^ tertile			Ref		Ref	
<60 min/d+2^nd^ tertile			0.35 (−1.68–3.38)		0.14 (−1.86–2.13)	
<60 min/d+1^st^ tertile			0.48 (1.81–2.76)		−0.75 (−3.00–1.49)	
≥60 min/d+3^rd^ tertile			**3.59 (0.44–6.74)**		**3.61 (0.39–6.82)**	
≥60 min/d+2^nd^ tertile			−0.69 (−3.43–2.05)		−1.07 (−3.83–1.68)	
≥60 min/d+1^st^ tertile			−0.01 (−2.74–2.72)		0.22 (−2.53–2.98)	
**Intraclass correlation coeficient**	0.007	0.06			0.10	0.05
**Standard deviation context**	3.42	2.87			4.03	2.57
**Standard deviation individual**	12.75	11.4			11.88	10.92
**Akaike Information Criterion**	13.442.2	4123.9			10,978.2	4,115.1

Beta coefficient and their respective confidence intervals 95% (95% CI).

* This analysis was adjusted for potential confounders: *age, socioeconomic status, parental education, regular tobacco smoking, body mass index and waist circumference.*

** 622 girls were excluded because they did not meet the inclusion criteria.

MPA = moderate physical activity.

VPA = vigorous physical activity.

MVPA = moderate and vigorous physical activity.

PA = physical activity.

SB = sedentary behavior.

Significant associations are in bold.

**Table 4:** Multiple linear regression analysis evaluating the association between systolic blood pressure according to independent variables for each study, in boys.

Independents Variables	Null model		Unadjusted		Adjusted*	
	HELENA	BRACAH	HELENA	BRACAH	HELENA	BRACAH
	ß (95% CI)	ß (95% CI)	ß (95% CI)	ß (95% CI)	ß (95% CI)	ß (95% CI)
**Fixed Effects Constant**	124.81 (124.10–125.51)	119.64 (118.42–120.86)			84.4 (76.1–92.7)	
**Physical activity by questionnaire**			p = 0.005	p = 0.331	p = 0.066	
≥60 min/d			Ref	Ref	Ref	
<60 min/d			**2.92 (0.90–4.96)**	**1.41 (−1.44–4.27)**	2.08 (−0.14–4.29)	
**Sedentary behavior by questionnaire**			p = 0.435	p = 0.811	p = 0.361	
<2 h/d			Ref	Ref	Ref	
2–4 h/d			1.18 (−0.77–3.14)	−1.32 (−7.77–5.14)	1.02 (−1.01–3.05)	−0.96 (−7.75–5.84)
>4 h/d			0.37 (−1.59–2.33)	−1.84 (−7.93–4.24)	−0.79 (−2.87–1.30)	−2.04 (−8.42–4.34)
**Physical activity by accelerometers** **			p = 0.399		p = 0.762	
≥60 min/d			Ref		Ref	
<60 min/d			0.74 (−0.99–2.47)		−0.30 (−2.21–1.62)	
**MPA by accelerometers (min/d)**			p = 0.895		p = 0.859	
		0.13 (−1.78–2.04)		−0.01 (−0.05–0.07)		
**VPA by accelerometers (min/d)**			p = 0.133		p = 0.929	
		−0.05 (−0.11–0.01)		−0.01 (−0.06–0.07)		
**MPA by accelerometers (min/d)**			p = 0.646		p = 0.936	
		−0.01 (−0.05–0.03)		−0.01 (−0.04–0.04)		
**SB by accelerometers (min/d)**			p = 0.069		p = 0.337	
		0.01 (−0.01–0.02)		0.01 (−0.01–0.02)		
**Cluster PA and SB by accelerometers** *			p = 0.673		p = 0.465	
<60 min/d+3^rd^ tertile			Ref		Ref	
<60 min/d+2^nd^ tertile			−0.58 (−4.09–2.94)		−2.16 (−5.49–1.16)	
<60 min/d+1^st^ tertile			0.36 (−3.14–3.85)		−0.09 (−3.37–3.18)	
≥60 min/d+3^rd^ tertile			0.88 (−2.56–4.31)		0.58 (−2.65–3.81)	
≥60 min/d+2^nd^ tertile			0.08 (−3.06–3.21)		0.30 (−2.65–3.24)	
≥60 min/d+1^st^ tertile			−0.88 (−3.75–1.98)		−0.06 (−2.86–2.74)	
**Intraclass correlation coeficient**	0.07	0.06			0.10	0.04
**Standard deviation context**	3.69	3.2			4.58	2.84
**Standard deviation individual**	13.98	13.2			13.91	13.40
**Akaike Information Criterion**	12,191.3	3,560.7			10,520.4	3,347.0

Beta coefficient and their respective confidence intervals 95% (95% CI).

* This analysis was adjusted for potential confounders: *age, socioeconomic status, parental education, regular tobacco smoking, body mass index and waist circumference.*

** 420 boys were excluded because they met the inclusion criteria.

MPA = moderate physical activity.

VPA = vigorous physical activity.

MVPA = moderate and vigorous physical activity.

PA = physical activity.

SB = sedentary behavior.

Significant associations are in bold.

**Figure 1:**
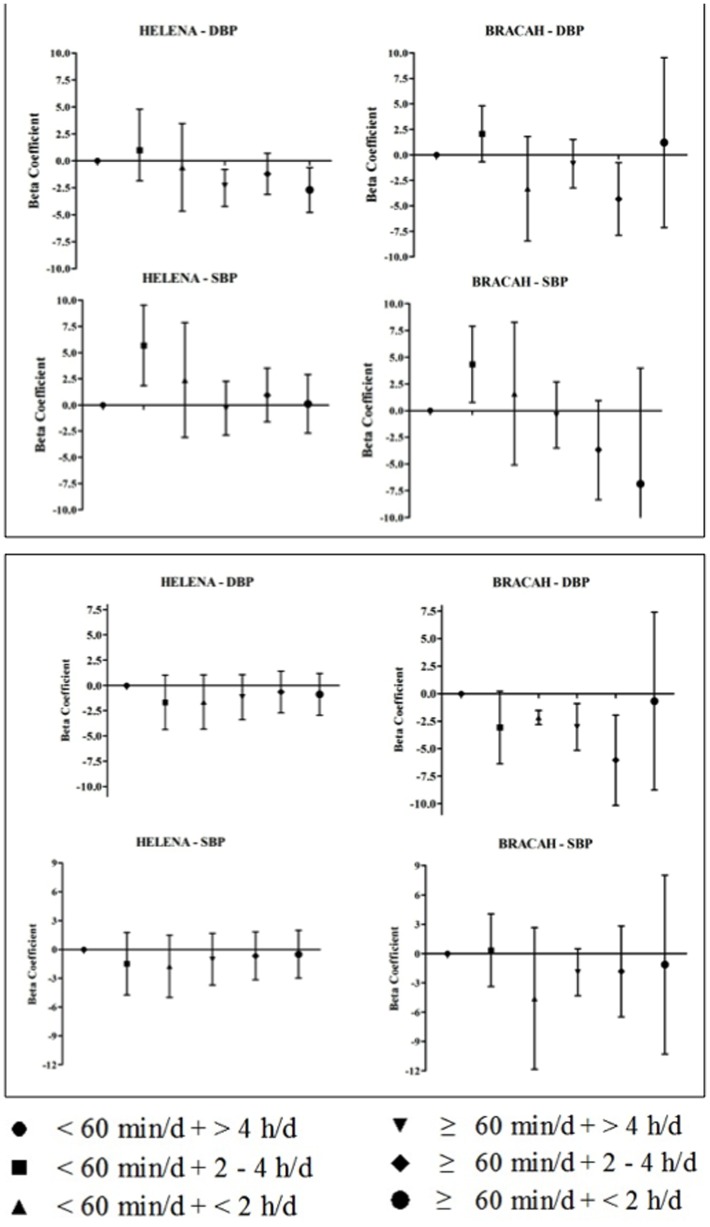
Beta coefficient and confidence intervals of 95% used to evaluate the association between blood pressure levels and clusters of physical activity and sedentary behavior measured by questionnaire in each study, (A) boys and (B) girls.

Figure 1 shows β coefficients and their respective 95%CI, evaluating the association between blood pressure levels and clusters of PA and SB measured by questionnaire for each study according to sex (A for boys; B for girls). After conducting the adjusted analysis, the PA and SB cluster levels: <60 min/d+<2 h/d **β = −2.17 (95%CI = −2.81–−1.53)**; ≥60 min/d+>4 h/d **β = −3.02 (95%CI = −5.14–−0.90)**; and ≥60 min/d+2–4 h/d **β = −6.05 (95%CI = −10.16–−1.94)** remained significantly and negatively associated with DBP in the girls from the BRACAH study.

In both studies, a significant association between diastolic BP, PA and SB cluster was observed in boys. In the HELENA study, the significant associations were observed in these categories in the PA and SB clusters: ≥60 min/d+>4 h/d **β = −2.31 (95%CI = −4.23–−0.39)**; and ≥60 min/d+<2 h/d **β = −1.21 (95%CI = −3.11–−0.71)**. While in the BRACAH study, a significant association was observed in this category: ≥60 min/d+2–4 h/d **β = −4.33 (95%CI = −7.90–−0.76)**. Moreover, the “<60 min/d +2–4 h/d” cluster was directly associated with systolic BP in both studies, with the largest effect presented by HELENA study adolescents (Figure 1).

## Discussion

The effect of PA and SB levels, both independently and combined, on the BP of adolescents from two observational studies was explored. The results suggest that meeting PA recommendations could mediate the effect of SB on diastolic BP in both genders in the BRACAH study and in boys from HELENA study; on the other hand, low PA plus excessive SB was associated with increases in systolic BP in boys. These findings were consistent in two different epidemiological studies conducted on adolescents, which strengthens the conclusions. Including data from two different studies adds consistency to our report as some of the results were similar in different populations (Hill's principles) [35].

Differences between studies might be explained by 1) associations between behaviors analysed and determinants of BP (culture, income, environment, among others) and 2) susceptibility of individuals to different social environments and cultures [36], [37]. The power remained greater than 90% in both studies, greater than 80% in the sex-specific analyses in both studies.

Our results differ from those of Ekelund et al. (2006), who found no differences in BP levels in the PA and SB cluster measured by accelerometers. On the other hand, our results corroborate studies that have evaluated the effects of PA and SB separately on BP levels [38], [39]. Several mechanisms can explain the positive effects PA induces on BP levels. There is strong evidence that the sheer stress caused by regular PA has a powerful effect on the release of vasodilator factors produced by the vascular endothelium [40], such as nitric oxide and endothelium-derived hyperpolarizing factor (EDHF) [41].

We observed that boys with low PA and high SB levels showed higher levels of systolic BP. This finding agrees with results from others studies in which adolescents with low PA and high SB presented low levels of cardiorespiratory fitness [42]. Low levels of PA, however, are also associated with other cardiovascular risk factors [43], [44]. In a recent review study, Pedersen and Febbraio [45] describe how SB (i.e., reduced muscle contractions) leads to an altered myokine response in skeletal muscle. Consequently, these alterations promote increased pro-inflammatory adipokines that may contribute to the development of endothelial dysfunction in the cardiovascular system (i.e., increased synthesis of interleukin-6 and pathological processes of atherosclerosis); these dysfunctions may progressively develop into hypertension. Nevertheless, there are several such possible physiological mechanisms by which PA and SB may contribute to increased BP, and more research is needed to analyze the pathophysiological processes of increased BP due to insufficient PA combined with SB.

Furthermore, in our European female sample, we found that adequate PA levels do not attenuate the effect of high levels of SB on systolic blood pressure, probably because: 1) females have reduced PA levels and 2) estrogens have a more powerful influence on BP levels than PA during adolescence [46].

Accelerometers allow the study of activity patterns, and can establish the dose-response relationship between activity and health outcomes. On the other hand, self-reported PA data may not accurately reflect activity patterns due to recall bias and/or social desirability bias [47]. Moreover, studying sedentary behavior can be exceptionally challenging. Questionnaires that use a single sedentary activity (like TV viewing) may be considered a somewhat one-dimensional way of estimating a rather broad spectrum of sedentary activities, and this approach does not estimate the broad range of sedentary behaviors that adolescents have. However, PA questionnaires have advantages over accelerometers, such as low cost and PA domain information.

Our results are of importance since the behavioral patterns under consideration during adolescence tend to continue into adulthood [48] and high levels of sedentary behavior in adults increase the risk of mortality from cardiovascular diseases [49], [50].

Since there were some methodological differences between the HELENA and the BRACAH studies (e.g., age range; accelerometers; geographic region) data from both studies were analyzed separately, but we used the multilevel analysis in order to control the influence of contextual (country-specific) variables, since several studies have shown that they influence PA and SB patterns [51], [52].

A limitation of this study is its cross-sectional design; consequently, causality cannot be established. Moreover, it was not possible to adjust the analysis for other potentially BP-associated factors in either of the two samples, such as genetics or intrauterine development, but we developed an adjusted analysis for large potential confounders. On the other hand, the diverse geographic origin of the samples, the use of objective measures to assess PA and SB and multilevel adjusted analysis are some of the main strengths of our study.

## Conclusions

According to our results, meeting current PA recommendations could mediate the association between SB and DBP in both sexes. In boys, the joint effect of low levels of PA and excessive sedentary activity increases SBP levels. These results suggest that regular PA should be promoted and SB discouraged in adolescent populations to prevent elevated blood pressure and its consequences in adulthood.

## Supporting Information

Table S1 **Characteristics of the samples from the HELENA study and the BRACAH study.**
(DOC)
