# Correction: Independent and Combined Effects of Physical Activity and Sedentary Behavior on Blood Pressure in Adolescents: Gender Differences in Two Cross-Sectional Studies

**DOI:** 10.1371/annotation/d02f73f9-3ab4-4bae-b25b-aa29e55cd4cf

**Published:** 2013-05-14

**Authors:** Augusto César Ferreira de Moraes, Heráclito Barbosa Carvalho, Juan Pablo Rey-López, Luis Gracia-Marco, Laurent Beghin, Anthony Kafatos, David Jiménez-Pavón, Dénes Molnar, Stefaan De Henauw, Yannis Manios, Kurt Widhalm, Jonatan R. Ruiz, Francisco B. Ortega, Michael Sjöström, Angela Polito, Raquel Pedrero-Chamizo, Ascensión Marcos, Frederic Gottrand, Luis A. Moreno

There was an error in the Funding statement.

The correct statement is: The HELENA Study takes place with the financial support of the European Community Sixth RTD Framework Programme (contract FOOD-CT-2005-007034). The writing group takes sole responsibility for the content of this article. This study was also supported by a grant from the Spanish Ministry of Health: Maternal, Child Health and Development Network (number RD08/ 0072), grant from the Spanish Ministry of Education (EX-2008- 0641) and the Swedish Heart-Lung Foundation (20090635). The BRACAH survey received no funding. ACFM received a scholarship from the São Paulo State Research Foundation (FAPESP: proc. 2011/11137-1 and 2011/ 20662-2). Luis A. Moreno was given scholarship of visiting professor from Brazilian government by Science without Borders Program by CNPq (National Counsel of Technological and Scientific Development) and CAPES (Coordination of Improvement of Higher Education Personnel) (proc. 007/2012). The GENUD Research Group co-financed by the European Regional Development Fund (MICINN-FEDER). The funders had no role in study design, data collection and analysis, decision to publish, or preparation of the manuscript. 

