# Supplementary material for: Independent and Combined Effects of Physical Activity and Sedentary Behavior on Blood Pressure in Adolescents: Gender Differences in Two Cross-Sectional Studies
Source: PLoS One. 2013 May 1;8(5):e62006. doi: 10.1371/journal.pone.0062006 (PMC3641137; doi:10.1371/journal.pone.0062006)
Supplement: Table S1 — Characteristics of the samples from the HELENA study and the BRACAH study. (DOC) [file pone.0062006.s001.doc]

Table 1: Characteristics of the samples from the HELENA study and the BRACAH study.

| **Variables** | **Boys** | | **Girls** | |
| --- | --- | --- | --- | --- |
|  | **HELENA (n=1,580)** | **BRACAH (n=451)** | **HELENA (n=1,728)** | **BRACAH (n= 540)** |
|  | **mean or % (95% CI)** | **mean or % (95% CI)** | **mean or % (95% CI)** | **mean or % (95% CI)** |
| **Age (years)** | 14.8 (14.7 - 14.9) | 16.4 (16.3 - 16.5) | 14.7 (14.6 - 14.8) | 16.2 (16.2 - 16.3) |
| **Education father** |  |  |  |  |
| Lower education | 6.8 (5.5 - 8.2) | 6.4 (4.1 - 8.7) | 8.1 (6.7 - 9.5) | 8.1 (5.7 - 10.4) |
| Lower secondary education | 29.6 (27.1 -32.0) | 25.2 (-21.1 - 29.3) | 29.5 (27.5 - 32.2) | 26.7 (22.9 - 30.5) |
| Higher secondary education | 27.6 (25.2 - 30.0) | 47.0 (42.2 - 51.7) | 27.3 (25.1 - 29.7) | 45.5 (41.2 - 49.8) |
| University degree | 36.0 (33.4 - 38.6) | 21.3 (17.5 - 25.2) | 34.7 (32.2 - 37.2) | 19.8 (16.3 - 23.2) |
| **Education mother** |  |  |  |  |
| Lower education | 7.5 (6.1 - 8.9) | 6.4 (4.1 - 8.7) | 8.0 (6.6 - 9.4) | 8.4 (6.1 - 10.8) |
| Lower secondary education | 26.3 (23.9 - 28.7) | 25.0 (20.9 - 29.1) | 24.9 (22.7 - 27.2) | 24.2 (20.5 - 27.9) |
| Higher secondary education | 31.2 (28.7 - 33.7) | 42.7 (38.0 - 47.3) | 31.8 (29.4 - 34.2) | 43.0 (38.7 - 47.3) |
| University degree | 35.0 (32.5 - 37.6) | 25.9 (21.8 - 30.0) | 35.2 (32.8 - 37.7) | 24.4 (20.7 - 28.1) |
| **Socioeconomic status** |  |  |  |  |
| High | 33.5 (31.0 - 36.1) | 14.4 (11.1- 17.8) | 31.0 (28.6 - 33.4) | 14.2 (11.2 - 17.2) |
| Medium | 56.5 (53.9 - 59.2) | 81.7 (78.0 - 85.3) | 56.1 (53.5 - 58.7) | 78.5 (75.0 - 82.0) |
| Low | 10.0 (8.3 - 11.6) | 3.9 (2.1 - 5.7) | 12.9 (11.1 - 14.6) | 7.3 (5.1 - 9.5) |
| **Systolic Blood Pressure (mmHg)** | 126.4 (125.5 - 127.1) | 119.6 (118.4 - 120.9) | 117.3 (116.7 - 117.9) | 107.0 (106.0 - 108.0) |
| **Diastolic Blood Pressure (mmHg)** | 68.5 (68.0 - 69.0) | 69.0 (68.1 - 69.9) | 69.3 (68.8 - 69.8) | 67.1 (66.2 - 68.0) |
| **Physical activity by questionnaire** |  |  |  |  |
| < 60 min/d | 14.4 (12.5 - 16.3) | 68.6 (64.2 - 72.9) | 16.0 (14.1 - 17.9) | 68.9 (64.9 - 72.9) |
| ≥ 60 min/d | 85.6 (83.7 - 87.5) | 31.4 (27.1 - 35.8) | 84.0 (82.1 - 85.9) | 31.1 (27.1 - 35.1) |
| **Sedentary behavior by questionnaire** |  |  |  |  |
| > 4 h/d | 38.8 (36.2 - 41.5) | 75.0 (70.9 - 79.1) | 20.4 (18.3 - 22.5) | 84.5 (81.3 - 87.6) |
| 2 - 4 h/d | 39.1 (36.4 - 41.7) | 20.6 (16.8 - 24.4) | 36.3 (33.8 - 38.8) | 12.5 (9.6 - 15.3) |
| < 2 h/d | 22.1 (19.9 - 24.4) | 4.4 (2.4 - 6.3) | 43.3 (40.7 - 45.8) | 3.1 (1.6 - 4.6) |
| **Physical activity by accelerometers*** |  |  |  |  |
| < 60 min/d | 72.5 (69.9 – 75.1) |  | 27.5 (24.9 – 30.1) |  |
| ≥ 60 min/d | 56.7 (53.5 – 59.8) |  | 43.3 (40.2 – 46.5) |  |
| **Sedentary behavior by accelerometers*** |  |  |  |  |
| 3^rd^ tertil | 633.2 (625.1 – 641.2) |  | 623.1 (618.3 – 628.0) |  |
| 2^nd^ tertil | 542.5 (540.4 – 544.5) |  | 542.5 (540.7 – 544.2) |  |
| 1^st^ tertil | 450.7 (445.8 – 455.6) |  | 464.2 (460.9 – 468.9) |  |
| **Cluster PA and SB by questionnaire** |  |  |  |  |
| < 60 min/d + > 4 h/d | 6.5 (5.2 - 7.9) | 52.3 (47.6 - 57.0) | 2.9 (2.0 - 3.8) | 59.3 (55.1 - 63.5) |
| < 60 min/d + 2 - 4 h/d | 5.5 (4.3 - 6.8) | 13.1 (9.9 - 16.2) | 6.0 (4.7 - 7.2) | 7.7 (5.3 - 10.0) |
| < 60 min/d + < 2 h/d | 2.4 (1.5 - 3.2) | 3.2 (1.5 - 4.9) | 7.1 (5.8 - 8.5) | 1.9 (0.7 - 3.1) |
| ≥ 60 min/d + > 4 h/d | 32.3 (29.8 - 34.8) | 22.7 (18.8 - 26.6) | 7.1 (5.8 - 8.5) | 25.1 (21.4 - 28.9) |
| ≥ 60 min/d + 2 - 4 h/d | 33.5 (29.8 - 34.8) | 7.6 (5.1 - 10.1) | 30.3 (28.0 - 32.7) | 4.8 (3.0 - 6.6) |
| ≥ 60 min/d + < 2 h/d | 19.8 (17.6 - 21.9) | 1.1 (0.1 - 2.1) | 36.1 (33.7 - 38.6) | 1.2 (0.2 -2.1) |
| **Cluster PA and SB by accelerometers*** |  |  |  |  |
| < 60 min/d + 3^rd^ tertil | 19.0 (16.5 – 21.4) |  | 27.8 (25.2 – 30.5) |  |
| < 60 min/d + 2^nd^ tertil | 12.2 (10.1 – 14.2) |  | 26.3 (23.7 – 28.9) |  |
| < 60 min/d + 1^st^ tertil | 12.2 (10.1 – 14.2) |  | 18.4 (16.1 – 20.6) |  |
| ≥ 60 min/d + 3^rd^ tertil | 12.6 (10.5 – 14.7) |  | 7.0 (5.5 – 8.4) |  |
| ≥ 60 min/d + 2^nd^ tertil | 17.7 (15.3 – 20.1) |  | 10.0 (8.3 – 11.8) |  |
| ≥ 60 min/d + 1^st^ tertil | 26.4 (23.6 – 29.1) |  | 10.5 (8.7 – 12.3) |  |
| **Regular smoking** |  |  |  |  |
| Yes | 12.2 (10.5 - 14.0) | 6.4 (4.1 - 8.7) | 12.8 (11.1 - 14.5) | 5.2 (3.3 - 7.1) |
| No | 87.8 (86.0 - 89.5) | 93.6 (91.3 - 95.9) | 87.2 (85.5 - 88.9) | 94.8 (92.3 - 96.7) |
| **Waist Circumference (cm)** | 74.4 (73.9 - 74.9) | 80.4 (79.5 - 81.4) | 70.2 (69.8 - 70.6) | 77.3 (76.5 - 78.1) |
| **Body Mass Index (kg/m2)** | 21.5 (21.3 - 21.7) | 21.8 (21.5 - 22.2) | 21.3 (21.1 - 21.5) | 21.2 (21.0 - 21.6) |

* Assessed only HELENA study; and 1042 adolescents were excluded because they did not meet the inclusion criteria.

PA= physical activity.

SB= sedentary behavior.
